# New Orleans Odontological Society

**Published:** 1886-05

**Authors:** C. Edmund Kells

**Affiliations:** Secretary


### NEW ORLEANS ODONTOLOGICAL SOCIETY.

REGULAR MONTHLY SESSION, DECEMBER 8, 1885.

REPORTED FOR THE INDEPENDENT PRACTITIONER BY C. EDMUND KELLS, JR., SECRETARY.

Dr. Chas. Eckhardt, President, in the chair.

The essayist of the evening, Dr. Geo. J. Friedrichs, being called upon, presented a paper on Aqua Calcis. (See page 235).

DISCUSSION.

*Dr. Jos. Bauer*—Had the pleasure of traveling last summer with the author of the paper so ably criticised, when that subject was brought up, and asked him for any physiological reason sustaining his points, but he could not give one; yet he claimed good results. Personally, the speaker had practiced in the alluvial districts of South-west Louisiana, and there found as good teeth as in regions 150 miles further north. Observation has long since taught him that the condition of the teeth depends more upon the general health than upon the local surroundings. As a proof of this, he cited the well known fact that the teeth of city people are poorer than are those of the country. There is an abundance of lime in the food we eat; the trouble lies in the assimilation. In the language of Dr. Bogue, "Thousands die of overeating, but none of starvation," to which I will add—at least not in Louisiana. During the meeting in Minneapolis this subject came up, and there seemed to be but two men in favor of lime water, one being the writer now criticised, and the other a *confrère* of his from St. Louis.

*Dr. A. G. Friedrichs*—The paper just read expresses my sentiments precisely. I believe that among the prominent predisposing causes for the production of defective dentures are hereditary influences. Again, the diseases of infancy through their influence on cell-nutrition, interfering with development of the teeth, is another cause. The inhabitants of cities, being much more exposed to these ills, are more affected than are the people of rural districts. As lime water is given to dissolve stone in the bladder, we might suppose, following the same line of reasoning, that taken medicinally it would not assist in development, but would, on the contrary, dissolve the lime out of the teeth.

*Dr. Geo. J. Friedrichs*—I would like to ask the speaker a question: Does he believe it possible for lime water to restore the parts of a tooth which have been lost by erosion or decalcification?

*Dr. A. G. Friedrichs*—Certainly not. It may, by the removal of external causes (keeping the saliva in an alkaline condition), tend to arrest disease, but never to restore the parts.

*Dr. John W. Adams*—Dr. J. D. White long ago wrote upon this subject, very much to the same effect as the paper which is receiving so much adverse criticism, but that was at a time when the merits of lime water were not so well understood as they are now. I was born in a limestone district, and I do not think the teeth here are inferior to those elsewhere.

*Dr. C. Edmund Kells, Jr.*—I have read with interest the late writers upon this subject, but my own experience has not given me any definite idea thereon, much as I have been interested in it. However, upon the principle that a man cannot grow fat and strong without food, I believe teeth cannot be well nourished without lime. I, therefore, have been using and do yet advise foods rich in lime salts. As for "Heredity," careful observation has led me to the opinion that we can place no reliance upon it. That the teeth of the generations now coming on are poorer than those of their parents, I am convinced, and I attribute the fact to the present general manner of living. I have many little patients, from twelve to fourteen years of age, who already have had more teeth filled than their parents. It has been suggested by some writer that excessive mental strain produces dental caries, and I have no doubt that the mental pressure under which children are now raised results in a general physical degeneration, and the teeth come in for their share of the trouble.

*Dr. Joseph Bauer*—With reference to the statement just made, that food is necessary to fatten a man, I find that when I go in the sugar region, during the grinding season, the fumes of the boiling juice make me fat. I have gained fifteen pounds during a rolling season. Children fed upon condensed milk are stouter than those fed upon ordinary food, but the tissue is not firm. The flesh gained upon a diet of cane-juice inhalations is the same. It is a well-known fact that consumptives, in the last stages of the disease, are brought to the sugar-houses, where they remain for a few weeks, during which time they get much stouter. The French are not great eaters; they eat a long time, but not very much.

*Dr. Geo. J. Friedrichs*—After all that has been said, we have drifted down to tissue function. Without the capability of the nutrient cells to appropriate what they need, it is folly to expect anything. If we had control of tissue-formation there would be no such thing as consumption, defective teeth, or, in fact, any of the ills to which flesh is heir, and the human race might live on indefinitely. Previous to the war, my preceptor, Dr. John S. Clark, who lived in Magnolia, Mississippi, had five friends, the finest specimens of manhood I ever saw, each weighing two hundred pounds at least, and standing six feet high in their stockings. They did not live in a limestone country. How could such osseous structures be produced in such a country, if lime water was essential as an element of development?

*Dr. P. J. Friedrichs*—I read to-day of a boy in lower Louisiana, seventeen years old and six feet ten inches tall. He must have gotten lime somewhere. Last winter, during the Exposition, I had occasion to examine the mouth of a St. Paul lady, whose brother was a dentist, and notwithstanding that she lived in a limestone district her teeth were full of fillings, and several crowns had been put on. If lime water is of any benefit, she should have had better teeth.

*Dr. Joseph Bauer*—I have a son who has never taken lime water (up to within a year, at least). Any hereditary tendency could have been nothing but bad—in regard to his teeth, I mean. Yet all his temporary teeth were sound, and at fourteen years of age but one or two crown fillings had been inserted.

*Dr. Geo. J. Friedrichs*—I wish to add the fact that many children, especially in cities, get more or less lime water, as it is given for indigestion, but at that time the permanent teeth are partly formed. Dr. Barrett's paper at the Minneapolis meeting pretty well disposed of the lime-water theory.

